# β-glycerophosphate, not low magnitude fluid shear stress, increases osteocytogenesis in the osteoblast-to-osteocyte cell line IDG-SW3

**DOI:** 10.1080/03008207.2024.2375065

**Published:** 2024-07-10

**Authors:** Robert Owen, Claudia Wittkowske, Damien Lacroix, Cecile M. Perrault, Gwendolen C. Reilly

**Affiliations:** a Department of Materials Science and Engineering, University of Sheffield, Sheffield, UK; b INSIGNEO Institute for In Silico Medicine, University of Sheffield, Sheffield, UK; c School of Pharmacy, University of Nottingham Biodiscovery Institute, University of Nottingham, Nottingham, UK; d Department of Mechanical Engineering, University of Sheffield, Sheffield, UK

**Keywords:** Matrix mineralization, mechanobiology, biomechanics, bone tissue engineering, extracellular matrix

## Abstract

**Aim:**

As osteoblasts deposit a mineralized collagen network, a subpopulation of these cells differentiates into osteocytes. Biochemical and mechanical stimuli, particularly fluid shear stress (FSS), are thought to regulate this, but their relative influence remains unclear. Here, we assess both biochemical and mechanical stimuli on long-term bone formation and osteocytogenesis using the osteoblast-osteocyte cell line IDG-SW3.

**Methods:**

Due to the relative novelty and uncommon culture conditions of IDG-SW3 versus other osteoblast-lineage cell lines, effects of temperature and media formulation on matrix deposition and osteocytogenesis were initially characterized. Subsequently, the relative influence of biochemical (β-glycerophosphate (βGP) and ascorbic acid 2-phosphate (AA2P)) and mechanical stimulation on osteocytogenesis was compared, with intermittent application of low magnitude FSS generated by see-saw rocker.

**Results:**

βGP and AA2P supplementation were required for mineralization and osteocytogenesis, with 33°C cultures retaining a more osteoblastic phenotype and 37°C cultures undergoing significantly higher osteocytogenesis. βGP concentration positively correlated with calcium deposition, whilst AA2P stimulated alkaline phosphatase (ALP) activity and collagen deposition. We demonstrate that increasing βGP concentration also significantly enhances osteocytogenesis as quantified by the expression of green fluorescent protein linked to Dmp1. Intermittent FSS (~0.06 Pa) rocker had no effect on osteocytogenesis and matrix deposition.

**Conclusions:**

This work demonstrates the suitability and ease with which IDG-SW3 can be utilized in osteocytogenesis studies. IDG-SW3 mineralization was only mediated through biochemical stimuli with no detectable effect of low magnitude FSS. Osteocytogenesis of IDG-SW3 primarily occurred in mineralized areas, further demonstrating the role mineralization of the bone extracellular matrix has in osteocyte differentiation.

## 1. Introduction

Bone strength is maintained through the continuous process of osteoblasts forming new bone tissue and osteoclasts breaking down existing bone matrix.^1,2^ This process is referred to as bone remodeling. Osteoblast and osteoclast activity are thought to be regulated via paracrine signaling from the highly mechanosensitive osteocytes which are located within the bone tissue.^3^ However, for osteoblasts to respond to these signals and deposit bone matrix, several other stimuli are also known to be important.

Biochemistry of the surrounding microenvironment governs bone formation. Ascorbic acid is known to be essential for the assembly of collagen fibers due to its acting as a co-factor for enzymes associated with collagen fibril assembly,^4,5^ whilst phosphate is necessary for hydroxyapatite deposition.^6^ The mechanical environment is also known to control bone biology. *In vivo*, bone cells are subjected to a wide range of mechanical forces, such as compression, strain, hydrostatic pressure, and fluid shear stress (FSS). FSS is induced by fluid flow within the porous bone matrix which is driven by small bone deformations due to gravitational and muscle forces^7^ and is widely regarded as a potent mechanical stimulus for bone cells.^8–11^ A relatively high wall shear stress of approximately 0.8 Pa to 3 Pa is predicted to be generated during physical activity within the microporous lacunar-canalicular system where the osteocytes are located;^12^ the effect of FSS on this highly mechanosensitive cell type has been the focus of the majority of bone mechanobiology research in the past.^13–16^

Less is known about the effect of FSS on osteoblasts that are in regions with larger porosities, such as the trabeculae. Here, in comparison to osteocytes, the more open and varied geometry of the microenvironment and differences in cell morphology make it more difficult to estimate the type and magnitude of fluid flow osteoblast experience. However, it is assumed that in comparison to osteocytes, the magnitude of the FSS would be much lower and comparable to that of interstitial fluid outside the lacunar-canalicular network.^1^

Studying the immediate and short-term effects of FSS on osteoblasts *in vitro* can be undertaken with relative simplicity as responses typically happen within a short time frame, with the activation of intracellular calcium signaling occurring instantaneously at the onset of flow and signaling molecule release such as prostaglandins occurring within the hour.^17^ Assessment of osteocyte calcium signaling *in vivo* in response to mechanical load has also been achieved with multiphoton microscopy.^18^ *In vitro*, the magnitude and type of fluid flow have been identified as important regulators for these responses,^19–22^ and these signaling cascades are thought to then trigger other bone formation processes such as collagen deposition and mineralization. However, the mechanisms by which these immediate responses translate into the much slower osteogenic processes are less well understood.^23^

The difficulty of understanding the effects of FSS on bone formation *in vitro* is, in part, due to the challenge of maintaining controlled fluid flow over long periods of time (weeks). Microfluidic systems have previously been used,^24^ but over extended periods of time the channels become obstructed by proliferating, matrix-depositing osteoblasts, altering the FSS applied. Other flow application methods circumvent these technical challenges, for example the use of parallel-plate flow chambers which can be disassembled following flow application,^25^ or systems which consist of relatively large flow channels with a height in the millimeter instead of micrometer range.^26^ However, these approaches are demanding to set up and can only apply very low shear stresses, respectively. An alternative to these methods for long-term, dynamic cell culture is to grow cells in multi-well plates on a see-saw rocking platform.^27^ Compared to parallel-plate systems, this approach is easy to set up and allows high-throughput testing, although achievable FSS is lower (τ_max_~0.06 Pa) even when using the largest diameter well plates which generate the highest FSS. These challenges with applying FSS *in vitro* and the range of bone cell types used in studies mean that data on long-term osteogenesis under controlled fluid stimulation are limited and contradictory, with some studies reporting no change in mineralization,^28^ while others state a significant increase in collagen and mineral deposition in response to fluid flow.^27,29^

IDG-SW3 is a relatively recently developed cell line that allows the study of osteocytogenesis: the differentiation of osteoblasts to late osteocytes. Alternative cell lines for studying this transition, such as MLO-A5 or MLO-Y4, have limitations such as an absence of key osteocyte proteins or an inability to produce mineralized matrix.^30^ IDG-SW3 was developed with the aim of overcoming these limitations due to the importance of matrix mineralization in osteocytogenesis,^31^ and as such has the potential to be one of the most effective tools for studying osteocytogenesis *in vitro* currently available. They are derived from transgenic mice where the dentin matrix phosphoprotein-1 (Dmp1) promoter drives the expression of the green fluorescent protein (GFP) in osteocyte-like cells,^30^ allowing osteocytes to easily be identified in culture. Dmp-1 is a marker for osteocyte differentiation^32^ and is critical for proper mineralization of bone.^33^ The role of FSS in osteocytogenesis is of interest as it is hypothesized that enhanced bone formation also accelerates osteocytogenesis as previous research suggests that mineralization may be a trigger for osteoblast to osteocyte differentiation.^34–38^

This study aims to provide a better understanding of how osteoblasts are affected during extended (weeks) time periods by FSS mechanical stimulation using the see-saw rocking method. Culturing IDG-SW3 in this experimental setup allows any effects on both bone formation and osteocytogenesis to be revealed, and in addition to mechanical stimulation, the effects of biochemical and temperature stimulation on bone formation and osteocytogenesis are also characterized for the first time in this cell line.

## 2. Materials and methods

All materials were purchased from Sigma-Aldrich, UK, unless otherwise stated.

### 2.1. IDG-SW3 culture

The murine osteoblast-late osteocyte IDG-SW3 cell line (Kerafast, USA) was used for all experiments between passages 18 and 36. At 33°C in the presence of interferon-γ (IFN-γ), these cells express a temperature-sensitive mutant of a tumor antigen that induces continuous proliferation and immortalization, but at 37°C without IFN-γ they resume typical, non-cancerous, osteoblast/osteocyte behavior.^30^

Basal media (BM) consisted of minimum alpha medium with ultraglutamine and nucleosides (Lonza, UK, cat# BE12-169F) supplemented with 10 vol% fetal bovine serum (Labtech, UK (FBS)), 100 units/ml penicillin, and 100 μg/ml streptomycin. Cells were passaged according to supplier instructions in expansion media (EM) consisting of BM supplemented with 50 units/mL IFN-γ (Gibco, UK) at 33°C in a humidified incubator with 5% CO_2_.

For experiments, cells were plated below confluence (either 10,000 or 25,000 cells/cm^2^) in 0.1% gelatin-coated plates and maintained at 33°C in EM until confluent (day 0). Then, excluding experiments where effects of temperature were investigated, cells were transferred to a 37°C environment and culture medium was replaced with supplemented media (SM), consisting of BM supplemented with 5 mM β-glycerophosphate (βGP) and 50 μg/ml L-ascorbic acid 2-phosphate (AA2P) to permit bone formation and osteocytogenesis. As flow experiments were conducted outside the cell culture incubator (5% CO_2_), culture medium was further supplemented with 25 mM HEPES buffer to maintain constant pH at ambient CO_2_ conditions. Culture medium was changed for all experiments every 2 to 3 days.

### 2.2. Characterisation of the role of temperature and media composition

Due to the recent development of IDG-SW3 cells, there are a limited number of publications reporting their use (52 PubMed results June 2024). In comparison to other bone cell lines, they require unconventional culture temperatures and media formulations; therefore, the effects of these parameters were initially investigated. To characterize their effects on IDG-SW3 bone formation and osteocytogenesis, cells were maintained from day 0 at either 33°C in EM or SM or at 37°C in EM or SM for 21 days.

### 2.3. Biochemical stimulation

To assess the effects of biochemical stimuli on IDG-SW3, the concentrations of βGP and AA2P were varied in the SM applied on day 0 (Table 1). All conditions were compared to the control group which was cultured in our standard SM (50 μg/ml AA2P, 5 mM β-GP).^27,39–41^

**Table 1.** Experimental conditions for biochemical stimulation of IDG-SW3 with AA2P and βGP.

Condition	AA2P (µg/ml)	βGP (mM)
#1 (Standard)	50	5
#2	50	10
#3	50	4
#4	50	2.5
#5	50	0
#6	0	0

### 2.4. Mechanical stimulation

Cells were subjected to temporally and spatially varying oscillatory FSS 5 days a week using two different rocking frequencies on a see-saw rocker (Stuart, UK) from day 0. The maximum shear stress *τ* at the center of a 6-well plate when the plate was in a horizontal position was estimated using Equation 1:^42^

(1) $$\tau =\frac{\pi \mu \theta_{\max}}{2\delta^{2}T}$$

where *μ* is the fluid viscosity (10^−3^ Pa s), *θ*_*max*_ is the maximal tilt angle (7°), *δ* is the ratio of the fluid depth in the well to the well diameter, and *T* is the time for one cycle (in seconds). Rocking parameters were selected to achieve a “low” and “high” shear stress condition based on previous rocking studies.^27,43–47^ Each well was filled with 2 ml of standard SM supplemented with 25 mM HEPES buffer. This resulted in a fluid depth of 2.08 mm in circular 6-well plates with a diameter of 35 mm; *δ* was calculated to be 0.06. The lower rocking frequency of 0.7 Hz generated a maximum shear stress of 0.038 Pa at the base of the well and was applied for either 1 h or 5 h per day. The higher frequency of 1 Hz was applied for 1 h per day and generated a maximum shear stress of 0.054 Pa. Fluid flow experiments were performed in an egg incubator (Rcom, USA) placed on a rocker to maintain a constant temperature of 37°C. Static controls were cultured in standard SM supplemented with HEPES and placed in a non-rocked egg incubator for 1 h per day.

### 2.5. Mineralised matrix deposition

Calcium and collagen deposition were quantified by alizarin red S (ARS) and direct red 80 (DR80) staining, respectively, as previously described.^48^ Samples were fixed with 10% formalin for 20 min then washed three times with deionized water. ARS solution (1% w/v) was added to each sample and incubated for 1 h at room temperature to stain calcium. Excess ARS was removed, and samples washed with deionized water until wash water remained clear. Calcium deposits were imaged with a digital camera before quantification by destaining with 5% perchloric acid for 15 min on an orbital shaker at 100 rpm. Absorption of each sample was measured in triplicate at 405 nm in a clear 96-well plate in a microplate reader (Tecan, CH). Subsequently, samples were washed four times with deionized water before DR80 staining (1 w/v% in saturated picric acid) for collagen for 1 h. Excess DR80 was removed, and samples washed until wash water remained clear. Stained collagen matrices were imaged before quantification by destaining with 0.2 M sodium hydroxide and methanol in a 1:1 ratio for 15 min on an orbital shaker at 100 rpm. Absorption of the destain solution was measured in triplicate at 540 nm.

### 2.6. Assessment of collagen deposition with second harmonic generation imaging

For biochemical and flow studies, second harmonic generation (SHG) images of fixed cells at day 21 were obtained using a Zeiss LSM 510 Meta upright confocal microscope (Zeiss, DE) equipped with a tuneable (700 nm to 1060 nm) Chameleon Ti:sapphire multi-photon laser. Images were taken using an excitation wavelength of 800 nm and a 40× water immersion objective. SHG emissions were collected in the backward scattering direction filtered through a 10 nm bandpass filter centered on 400 nm.

### 2.7. Assessment of metabolic activity

PrestoBlue^TM^ Cell Viability agent (ThermoFisher, UK) was used to quantify cell metabolic activity according to manufacturer’s instructions. Briefly, media was replaced with PrestoBlue^TM^ working solution (10% PrestoBlue^TM^ in BM) and incubated at 37°C for 1 h. The solution was then transferred to a black 96-well plate and fluorescence detected at λex: 560 nm, λem: 590 nm in a microplate reader (Tecan Infinite 200, Switzerland), where fluorescence correlates with metabolic activity.

### 2.8. Total DNA and alkaline phosphatase activity quantification

DNA and alkaline phosphatase (ALP) were released from cell cultures by refrigerating overnight in cell digestion buffer (0.15 M Trizma® hydrochloride pH 8.8, 100 µM ZnCl_2_, 100 µM MgCl_2_, and 1 vol% Triton X-100). Wells were scraped using a cell scraper and the lysates transferred to a microcentrifuge tube before vortexing and freeze-thawing three times (10 min at −80°C, 15 min at 37°C). Lysates were then centrifuged at 10,000 g for 5 min before homogenizing the supernatant. Total DNA was determined with the Quant-it PicoGreen dsDNA assay kit (Life Technologies, UK) according to manufacturer’s instructions. Briefly, 10 µL of lysate was combined with 90 µL of substrate in a black 96-well plate. Plates were shaken to aid DNA-substrate conjugation, incubated for 10 min at room temperature, then shaken again before measuring fluorescence using a microplate reader (485 nm excitation, 520 nm emission, Tecan Infinite 200, Switzerland). ALP activity was quantified using the Pierce^TM^ PNPP substrate kit (ThermoFisher Scientific, UK) according to manufacturer’s instructions. Briefly, 20 μL of lysate was combined with 180 μL of substrate in a 96-well plate. The change in absorbance at 405 nm was recorded every 60 s for 30 min using a microplate reader (Tecan Infinite 200, Switzerland). Activity is expressed as nmol of p-nitrophenol per minute (nmol pNP/min), assuming one absorbance value equals 19.75 nmol of product. This activity was normalized to the amount of DNA to allow cellular level comparisons.

### 2.9. Assessment of osteocytogenesis

The transformation from osteoblast-like cells to osteocyte-like cells was assessed by measuring the expression of the fluorescent Dmp1-GFP marker. To determine GFP fluorescence in live cells, culture medium was exchanged for Hank’s Balanced Salt Solution (HBSS) before reading fluorescence in top reading mode at 36 locations per well using a microplate reader (485 nm excitation, 535 nm emission, Tecan Infinite 200, Switzerland) with the well plate lid removed. Control values from cell-free wells containing HBSS were subtracted from each reading. Exceptionally high or low readings caused by cell layer detachment were excluded from further analysis.

### 2.10. Microscopy

Phase-contrast and fluorescence images were obtained with a Ti-E Nikon inverted microscope (Nikon). GFP signal was detected using a filter system with 470 nm excitation, 525 nm emission, and a 2 s exposure time. Cell nuclei were imaged (350 nm excitation, 460 nm emission, 100 ms exposure) after fixed samples were stained for 30 min with 4,’6-diamidino-2-phenylindole (DAPI) solution (BD Biosciences) at a concentration of 5 µg/ml. Image analysis was performed with ImageJ.^49^

### 2.11. Statistical analysis

Data are expressed as mean ± standard deviation (SD). Number of replicates for each experiment are provided in the figure legends. Graphical representation and statistical analysis were performed using GraphPad Prism. One- or two-way analysis of variance (ANOVA) was performed depending on the number of independent variables, with Bonferroni’s multiple comparisons test used to identify significant differences. *p* < 0.05 (*) was considered significant, and *p* values < 0.01 are noted as **.

## 3. Results

### 3.1. Culturing at 33 °C holds IDG-SW3 in their osteoblastic phenotype

**Figure 1.**
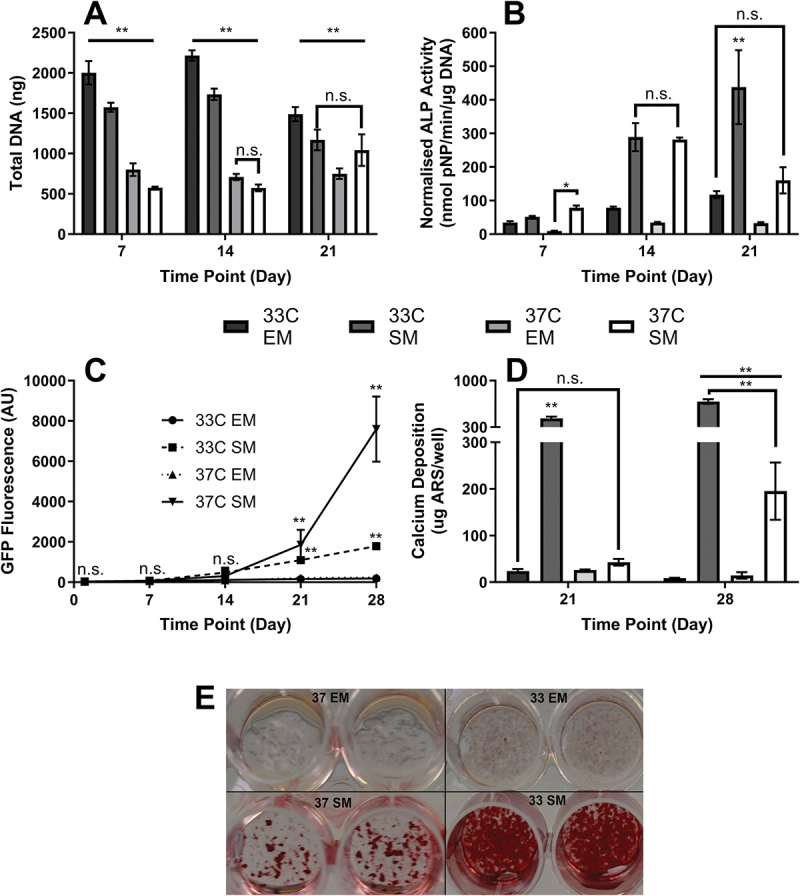
**Effect of culture temperature and media composition**. (a) Total DNA—all groups significantly different at all time points (*p* < 0.01) except day 14 37°C EM vs. 37°C SM and day 21 33°C SM vs. 37°C SM. (b) Normalised ALP activity—no significant difference between the 2 SM cultures on day 14. 33°C SM significantly higher than all other groups on day 21 (*p* < 0.01). No difference between 33°C EM and 37 °C SM on day 21. (c) Osteocytogenesis by GFP expression—No significant differences until day 21 when both SM cultures become greater than EM cultures onwards. 37°C SM highest at days 21 and 28. (d) Calcium deposition—significantly higher in 33°C SM at both time points than all other groups. 37°C SM calcium deposition only significantly greater than EM groups on day 28 (all *n* = 4). (e) Representative photograph of ARS staining on day 28. Well diameter 11 mm.

As expected, due to the thermal control of the tumor antigen, the greatest amount of DNA was observed in cultures maintained at 33°C in expansion media (33C EM), with 33C EM having 2.5× more DNA than cultures maintained at 37°C in expansion media (37C EM) on day 7 (Figure 1(a)). On days 14 and 21, there was no significant difference in DNA content at 37°C, regardless of media composition.

The highest normalized ALP activity occurred in cells cultured in supplemented media (SMs), with no significant additional effect of temperature in this media type on days 7 and 14. However by day 21, whereas cells maintained at 37°C in supplemented media (37C SM) underwent a decrease in ALP activity in comparison to day 14 (as associated with osteocytogenesis), cells maintained at 33°C in supplemented media (33C SM) saw an increase in normalized ALP activity in comparison to day 14 and had significantly higher ALP activity than cells maintained 37C SM, indicating they were being held in an osteoblastic phenotype (Figure 1(b)).

This observation agrees with direct osteocytogenesis quantification measured by GFP expression, which had no significant differences until days 21 and 28 when supplemented media cultures were significantly higher than expansion media cultures, and 37C SM was significantly higher than 33C SM at both time points − 4.25× greater on day 28 (*p* < 0.01) (Figure 1(c)).

Furthermore, this retention of osteoblast behavior was observed when determining calcium deposition, which was significantly higher than all other groups on both day 21 and day 28 in 33C SM, with 37°C SM only being significantly higher than EM cultures on day 28 (Figure 1(d,e)).

### 3.2. Omission of ascorbic acid reduced collagen deposition and ALP activity

**Figure 2.**
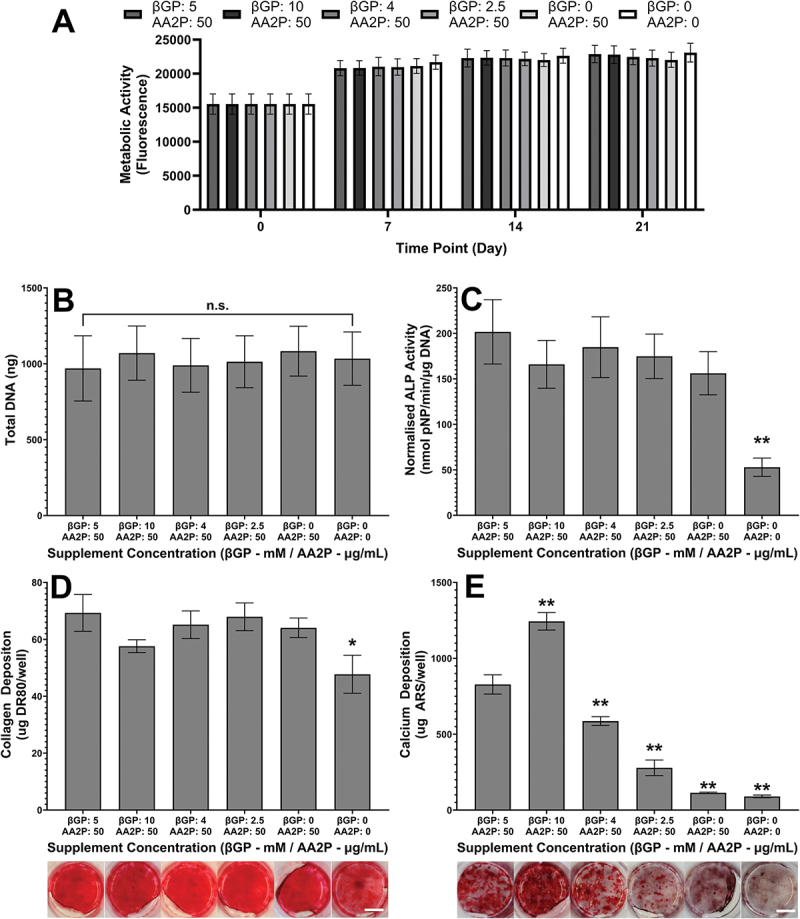
**Effect of biochemical treatment on mineralized matrix formation at day 21**. None of the biochemical treatments had a significant effect on (a) metabolic activity or (b) total DNA over a 21-day period. (c) Normalised ALP activity—omission of AA2P significantly reduced normalized ALP activity through a reduction in ALP expression. (d) Collagen deposition visualized and quantified by by DR80. Omission of AA2P resulted in significantly lower deposition of collagen. (e) Calcium deposition visualized and quantified by ARS. βGP dose-dependently enhanced mineralization. Significant differences are compared to standard condition (first column, 5 mM βGP, 50 µg/ml AA2P). *p < 0.05, **p < 0.01, *n* = 9. Scale bar = 7.5 mm.

None of the biochemical treatment parameters detailed in Table 1 had a significant effect on cell proliferation as determined by metabolic activity between day 0 and 21 (Figure 2(a)) and the total amount of DNA per well at day 21 (Figure 2(b)). Therefore, significant reduction in normalized ALP activity when AA2P was omitted from the media is a result of lower ALP expression rather than a change in cell number (Figure 2(c)). As expected, the amount of collagen deposited was very low unless ascorbic acid 2-phosphate was present (*p* < 0.05). Visual inspection of the DR80-stained samples confirmed that a layer of collagen was present within the wells, but varying βGP concentration had no effect on the amount deposited (Figure 2(d)).

### 3.3. Phosphate dose-dependently enhanced mineralisation

IDG-SW3 deposited significant amounts of mineral by day 21 as indicated by a strong ARS staining, with the amount of deposited mineral dependent on βGP concentration (Figure 2(e)). For example, doubling βGP concentration from 5 mM to 10 mM resulted in a 50% increase in mineral deposition (*p* < 0.01). In contrast, reducing βGP concentration resulted in significantly less mineralization (*p* < 0.01). Media without βGP did not support mineralization.

### 3.4. Osteocyte differentiation in IDG-SW3 is dependent on phosphate concentration and mineralisation

**Figure 3.**
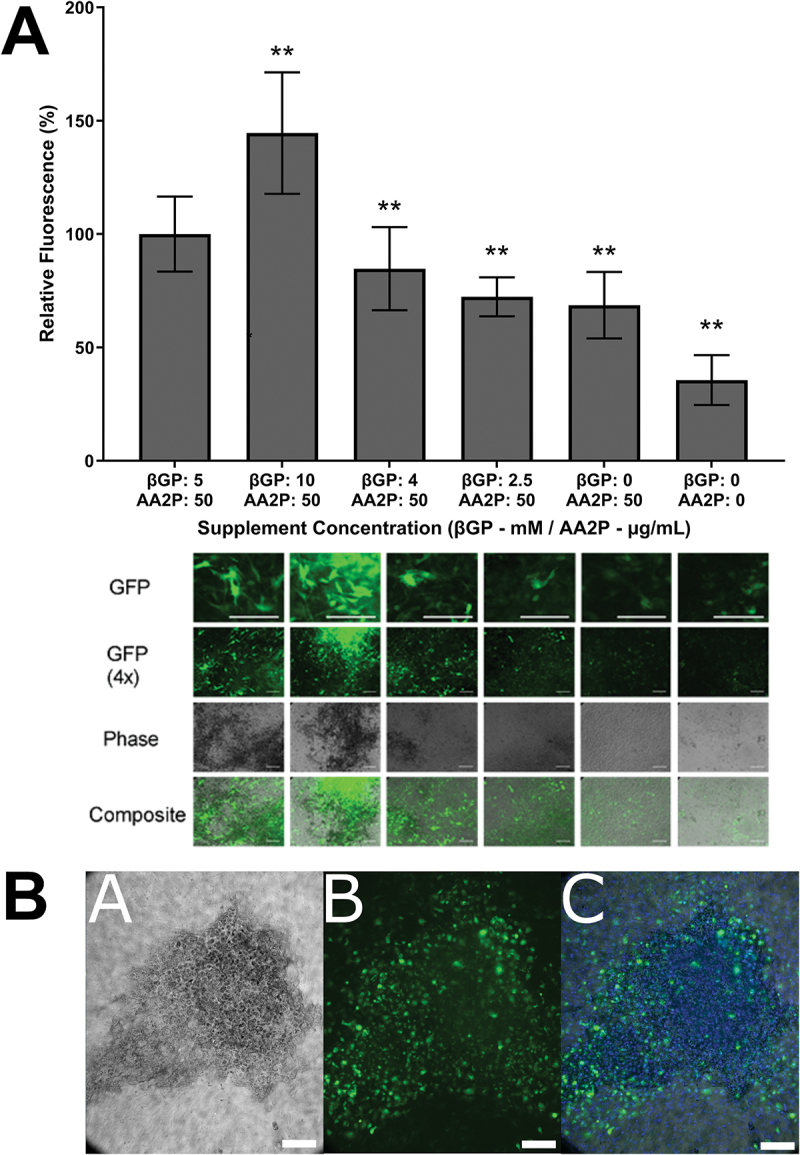
**Effect of biochemical treatment on osteocytogenesis at day 21**. (A—top) Dmp1-GFP expression compared to standard conditions (first column, 5 mM βGP, 50 µg/ml AA2P, **p < 0.01, *n* = 9). (A—bottom) Corresponding fluorescence microscope images (scale bars 250 µm). Dmp1-GFP positive cells (green), phase-contrast images show cell contours and mineralized areas in black. (b) Higher magnification image of a mineral nodule showing co-localization of (a) mineralized areas and (b) high concentrations of Dmp1-GFP positive cells. Composite (c) of phase contrast, GFP and nuclei (blue). Scale bars = 200 µm.

Expression of the osteocyte marker Dmp1, which is linked to GFP fluorescence in IDG-SW3, increased in all culture conditions from day 0 to day 21 (*p* < 0.05, data not shown). βGP concentration positively correlated with Dmp1 expression, with higher concentrations of βGP resulting in greater expression of GFP; 10 mM versus 5 mM βGP increased GFP fluorescence by 50% on day 21 (Figure 3(a), *p* < 0.01). In contrast, there was less GFP fluorescence from cells cultured with little or no βGP (*p* < 0.01), with the least GFP fluorescence detected when both βGP and AA2P were omitted (*p* < 0.01). The surface area covered with mineral was higher at greater βGP concentrations. Dmp1-GFP positive cells were primarily located in the mineralized areas, which also had a higher cell density than surrounding areas (Figure 3(b)).

### 3.5. There was no detectable effect of low fluid shear stress on proliferation or ECM deposition

**Figure 4.**
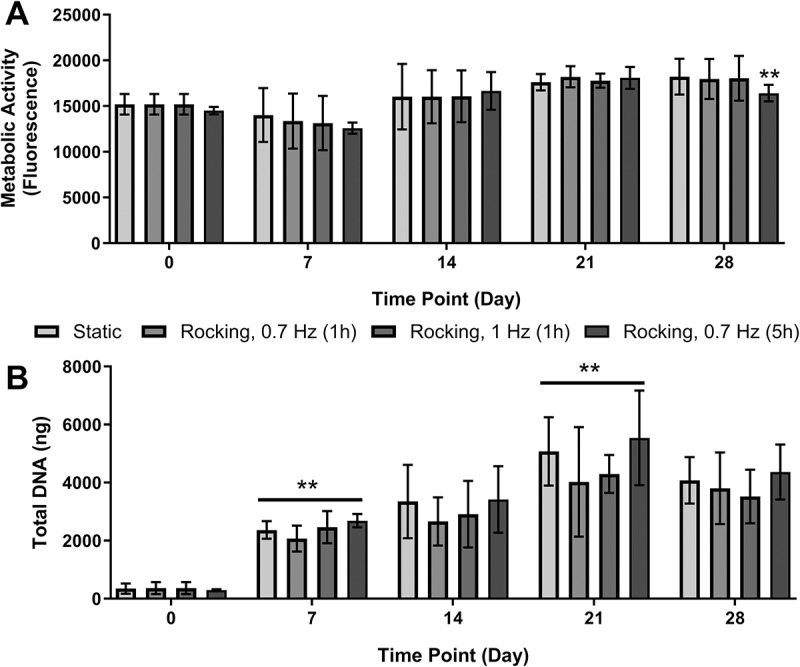
**Effect of low fluid shear stress on proliferation**. No significant difference in (a) metabolic activity between rocked and static samples at any time point except static vs 0.7 Hz/5 h at day 28. (b) Total DNA per sample increased from day 0 to day 21 then plateaued to day 28. No significant differences between static and rocked groups. Significant differences are compared to previous time point. **p < 0.01, *n* = 9.

No significant difference in proliferation was detected between static and mechanically stimulated samples at any time point as indicated by metabolic activity (Figure 4(a)) and total DNA (Figure 4(b)), except for the metabolic activity of 0.7 Hz/5 h rocked samples vs. static at day 28. Total DNA significantly increased from the beginning of culture up to day 21 (*p* < 0.01), with cell numbers plateauing between day 21 and day 28 (Figure 4).

**Figure 5.**
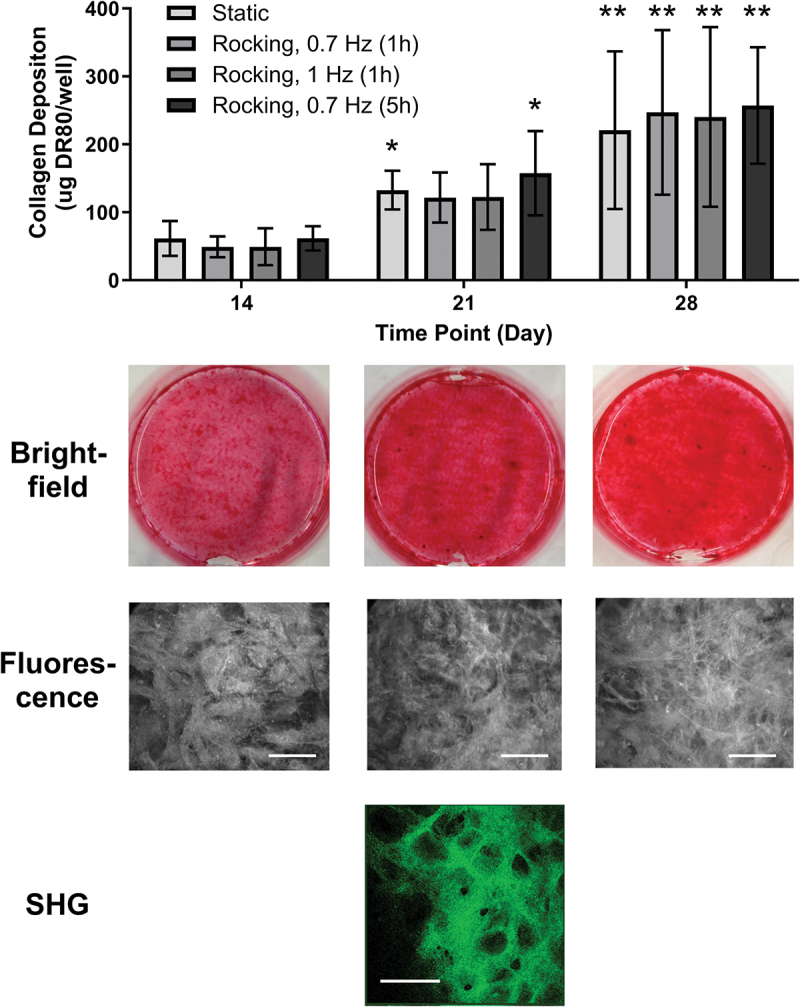
**Effect of low fluid shear stress on collagen deposition**. Top: Quantitative assessment of collagen deposition by DR80 destaining—no significant differences between static and rocked groups. The amount of collagen increased in all conditions from day 14 to day 28 (significant differences are compared to previous time point; *p < 0.05, **p < 0.01, *n* = 9). Bottom: Corresponding photographs of static samples. Brightfield images show red stained collagen fibers. Intracellular pro-collagen and extracellular collagen fibers can be visualized with red fluorescent light after staining with DR80. The amount of extracellular collagen fibers increased over time. Extracellular fibers of non-stained samples were visualized with second harmonic generation (SHG) imaging. Extracellular collagen networks formed honeycomb structures which were several micrometers thick. There were no differences in fiber or cell orientation between static and rocked samples. Scale bars = 50 µm.

In all conditions, collagen was deposited throughout the whole well and the amount of collagen increased significantly between day 14 and day 28 (Figure 5, *p* < 0.01). Mechanical stimulation at the levels applied did not result in greater collagen deposition at any of the observed time points. Fluorescence microscopy images of DR80 stained samples showed an increase in fiber density over time, thereby confirming quantitative results from DR80 destaining. The thickness of the collagen layer varied greatly between different locations in the same well and reached up to 20 µm as confirmed by SHG imaging. Cell and fiber orientation were not affected by any of the tested FSS regimes. Collagen fibers were arranged in honeycomb structures which appeared to be independent of the underlying cell orientation or the direction of fluid flow (Figure 5).

**Figure 6.**
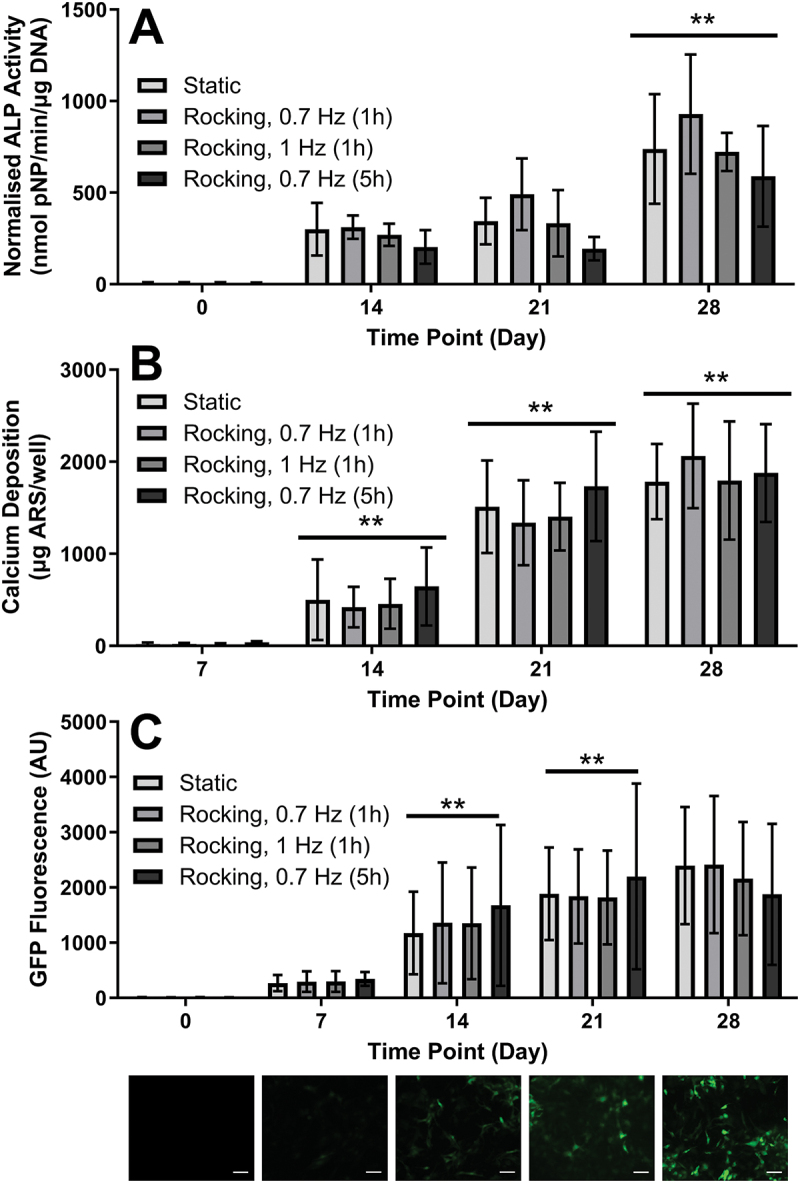
**Effect of low fluid shear stress on ALP activity, mineralization and osteocytogenesis**. (a) Normalised ALP activity increased over time. (b) Calcium deposition increased in all conditions from day 7 to day 28, with the highest increase between day 14 and day 21. For both ALP activity and mineralization, static and rocked conditions were not significantly different. (c—Top) Dmp1-GFP as an osteocyte marker increased significantly over time in all four conditions and was not significantly different between static and rocked samples. (c—Bottom) Corresponding representative fluorescence images from static sample. There were no significant differences in cell orientation, cell morphology and cell distribution between static and rocked samples. Significant differences are compared to previous time point. **p < 0.01, *n* = 9.

Normalized ALP activity increased in all conditions from day 0 to day 28 (Figure 6(a), *p* < 0.01). However, there was no significant difference in ALP activity between static and mechanically stimulated samples. Mineral deposition significantly increased between day 7 and day 21 in all conditions (Figure 6(b), *p* < 0.01), then plateaued between day 21 and day 28. The amount of mineral was negligible at day 7, with no detectable, red-stained mineral in any of the wells. Rocking did not significantly enhance mineralization compared to the static control group. ARS staining showed that mineral was distributed evenly in the wells.

### 3.6. There was no detectable effect of low fluid shear stress on osteocyte differentiation

**Figure 7.**
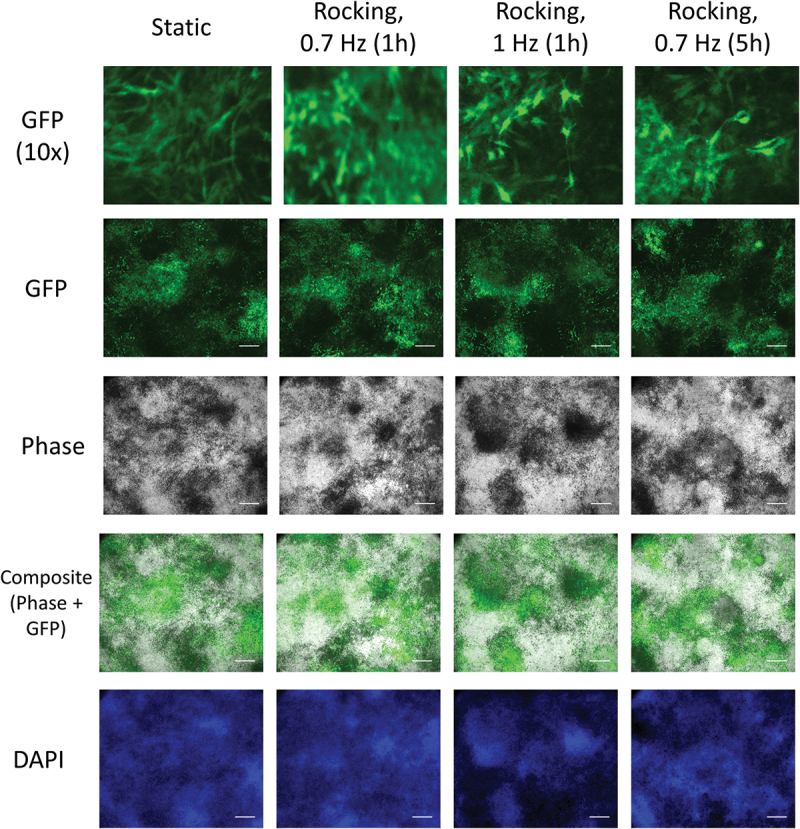
**Co-localization of mineral and Dmp1-GFP positive cells**. In all four conditions mineral deposition and Dmp1-GFP positive cells were co-localized. The black areas in the phase-contrast images are the mineralized areas. Dmp1-GFP positive cells exhibited a spindle-like morphology with processes. A higher concentration of DAPI-stained cell nuclei can be found in mineralized areas. All images were taken at day 28 and are representative ones from the center of a well of three independent experiments. Scale bar = 500 µm.

Dmp1-GFP fluorescence significantly increased between day 0 and 28 in all conditions (Figure 6(c), *p* < 0.01), but there was no significant difference between static and mechanically stimulated groups. Fluorescent images showed that Dmp1-GFP positive cells were primarily located in strongly mineralized areas. Nuclei staining (blue) further indicated that these areas also exhibited the highest concentration of cells. Dmp1-GFP positive cells had spindle-like morphology and often exhibited thin processes (Figure 7).

## 4. Discussion

The aim of this study was to investigate whether FSS enhances bone formation and osteocytogenesis in comparison to biochemical treatments using the osteoblast–osteocyte cell line IDG-SW3. However, unlike more common osteoblast-lineage cell lines such as MC3T3-E1, MLO-A5, and MLO-Y4, IDG-SW3 is cultured in atypical conditions to allow thermal and chemical control over a switch between their proliferating and differentiating state. As there are only a limited number of published studies on IDG-SW3 due to its relatively recent development, before exploring the influence of FSS, a characterization of how IDG-SW3 responds to these different temperature and chemical stimuli was performed.

IDG-SW3 is passaged at a lower-than-normal temperature (33°C) in the presence of interferon-γ (IFN- γ) to induce expression of a temperature-sensitive mutant of a tumor antigen that induces continuous proliferation and immortalization. When returned to standard culture conditions (37°C, without IFN- γ), this mutant is no longer expressed and IDG-SW3 resumes their normal osteoblast–osteocyte transition. Consequently, it was examined how these two factors (temperature, presence of IFN- γ) affect their osteoblast activity and osteocytogenesis. As expected, the highest cell numbers were observed when cultures were maintained at 33°C due to the temperature-sensitivity of the SV40 antigen used to control proliferation. Interestingly, even though this mechanism is intended to be controlled by the synergy of the lower temperature and presence of IFN-γ, significantly more cells were present at 33°C in IFN-γ lacking media than the 37°C cultures, indicating continuous proliferation is still induced at the lower temperature in the absence of IFN-γ, albeit at a lower level.

Significantly lower ALP activity was observed in expansion media (EM) cultures regardless of temperature, indicating the importance of βGP and AA2P in the supplemented media (SM) cultures for normal osteoblastic behavior of IDG-SW3. When maintained at 33°C in SM, ALP activity significantly increased at each time point indicating IDG-SW3 cells were being held in an osteoblastic phenotype rather than undergoing osteocytogenesis. In contrast, when maintained at 37°C in SM, ALP activity peaked between day 14 and 21, reducing in the final week as osteocytogenesis proceeded. This is corroborated by Dmp1-GFP fluorescence, with only the SM cultures significantly increasing in fluorescence over time, and the expression in the optimal 37°C supplemented media condition for osteocytogenesis increasing exponentially to levels significantly higher than all other groups.

Unsurprisingly, significant mineralization only occurred in SM groups, but interestingly and again supporting that the osteoblast phenotype is retained at 33°C rather than undergoing osteocytogenesis, much greater calcium deposition occurred at the reduced culture temperature than 37°C. The greater mineralization is likely a result of at least two factors: first, increased proliferation at the lower temperature resulting in earlier confluence and therefore osteoblasts depositing mineralized matrix at an earlier time point in the 33C SM condition, and second, that the greater osteocytogenesis at 37C SM results in a greater number of sclerostin-expressing osteocytes that inhibit mineralization.^30,50^ In summary, IDG-SW3 appears to only be able to undergo osteocytogenesis when maintained in conditions that permit mineralized matrix deposition (supplemented media). When maintained in these conditions, mineralized matrix deposition and osteocytogenesis can occur at both 33°C and 37°C, although the greatest matrix deposition occurs at the lower temperature and the greatest osteocytogenesis at the higher temperature. Importantly, despite the requirement for mineralization to occur for osteocytogenesis to be possible, there are diminishing returns; forcing excessive mineralization through 33°C culture does not result in superior osteocytogenesis, instead having an inhibitory effect in comparison to 37°C.

With it established that mineralization is essential for osteocytogenesis to occur, the influence of varying AA2P and βGP concentration on mineralized matrix deposition of IDG-SW3 was investigated. Whilst they had no significant effect on proliferation (Figure 2(a,b)), the deposition of significantly less collagen, lower ALP activity, and negligible mineralization in the absence of AA2P in comparison to AA2P replete media confirmed the vitamin’s essential role in osteogenesis (Figure 2(c,e)). It is required for proper collagen assembly,^4,5^ and there is evidence that induction of the osteoblast phenotype and subsequent ALP expression is dependent on the interface between osteoblasts and a collagen-containing extracellular matrix.^51^

With AA2P present, ALP activity was observed to be independent of βGP concentration (Figure 2(c)), although increasing phosphate concentrations significantly increased mineralization (Figure 2(e)), with the greatest calcium deposition observed at 10 mM. The increased mineralization caused by higher concentrations of βGP also enhanced osteocytogenesis (Figure 3(a)), with the greatest number of osteocytes occurring in the most mineralized cultures. Interestingly, the spatial distribution of osteocytes within the cultures overlays almost perfectly onto the regions where mineralized nodules are deposited by the cells (Figures 3(b), 7). This observation indicates that the osteocytogenesis of IDG-SW3 *in vitro* resembles the requirements for osteocytogenesis *in vivo*, where it is currently widely accepted that osteoblasts are self-buried or embedded by neighboring cells into the mineralizing osteoid and as a result experience an environmental stimulation that commits them to becoming osteocytes rather than the other termini of the osteoblast lineage (bone lining cell or apoptosis).^31^ Although a βGP concentration of 10 mM gave the greatest osteocytogenesis here and this concentration is often utilized in osteogenic media formulations,^52–54^ it has been shown that such high concentrations can result in abnormal, non-osteoblast mediated mineralization caused by spontaneous precipitation of calcium phosphates.^55^ Therefore, for subsequent studies investigating the influence of FSS, concentrations of 5 mM were used.

Application of low magnitude FSS using a see-saw rocker had no stimulatory or inhibitory effect on proliferation in comparison to static cultures (Figure 4), which is in line with previous work.^27^ Regardless of culture regimen, cell number (total DNA) increased during the first 3 weeks of culture. Since day 0 was defined as the time point when cells reached confluence and therefore IDG-SW3 was transferred to 37°C and SM and FSS were applied, increasing cell number meant that cells tended to form high-density clusters. Continuous proliferation should cease when cultured at 37°C in the absence of INF-γ;^30^ however, as observed in Figure 1, some residual proliferation does still occur during the experimental period. This may be the result of a non-immediate deactivation of the tumor antigen that controls proliferation, and as such it can be difficult to tightly control the starting cell number between experimental repeats or laboratory users due to the arbitrary nature of visual estimation of confluence in cell culture.

There was no detectable effect of the applied flow regime on osteogenesis, with no significant changes in collagen production, ALP activity, or calcium deposition of IDG-SW3 (Figure 5, 6). For collagen synthesis, this was not unexpected as there is conflicting information on whether collagen deposition is stimulated under flow, with studies investigating mesenchymal stromal cells^24,56^ or the post-osteoblast cell line MLO-A5.^27^ indicating minor positive effects, whilst other research has not been able to demonstrate an anabolic effect of flow on collagen at either the mRNA^14^ or deposited protein level.^44,47^ The application of FSS using see-saw rocking not affecting ALP activity is also consistent with the work of Puwanun et al., which found no effect on the ALP activity of human embryonic derived mesenchymal progenitors, hES-MPs.^44^ Whilst mineralized matrix deposition is the parameter most frequently reported to be upregulated by this type of mechanical stimulation on other osteoblast-lineage cell types,^27,44,57^ a positive effect is not ubiquitous. In separate work with the same rocker system and comparable shear stresses, MLO-A5 and MC3T3-E1 were shown to also not respond to these levels of FSS, and hES-MPs to a lesser extent than studies using different rocking platforms unless mechanosensitivity is increased through lengthening of the primary cilium via application of lithium chloride.^47^

Considering the close relationship observed thus far between IDG-SW3 mineralization and osteocyte differentiation, it follows that with no enhanced calcium deposition in response to FSS, no effect on osteocytogenesis was observed either. Despite this, in all conditions IDG-SW3 differentiated toward osteocyte-like cells over time as confirmed by increased expression of the osteocyte-marker Dmp1.^30,58^ We have shown that this temporal change in IDG-SW3 osteocytogenesis is affected by the biochemical environment, although it was not possible to distinguish whether it is the increased mineralization resulting from raised βGP concentration that triggered osteocyte differentiation,^36^ or if the greater phosphate concentrations directly stimulated osteocytogenesis.^59^ Recent work has shown that inorganic phosphate (Pi) up to concentrations of 1.5 mM released from octacalcium phosphate materials directly stimulated osteocytogenesis in IDG-SW3, but concentrations above this were inhibitory.^60,61^

The lack of detectable response of IDG-SW3 cells to the mechanical stimulation applied here was unexpected due to the widely acknowledged role of osteoblast-lineage cells and especially osteocytes as mechanoresponsive cells. From this study, it seems apparent that osteocytogenesis of IDG-SW3 is coupled to mineralization, and the main driver of this process is the physical entrapment of the cells within osteoid. The spatial coupling of mineralization to osteocyte formation means it is unlikely that a flow profile could be identified that directly stimulates this terminal differentiation *in absentia* of mineralized osteoid regardless of the magnitude of FSS. However, it is feasible that a different magnitude or methodology of applying FSS to IDG-SW3 could further stimulate mineralized matrix deposition in line with how high concentrations of βGP achieved greater mineralized matrix deposition.

The low magnitude FSS applied in this study may have been below the threshold required to stimulate IDG-SW3; however, the applied magnitude was intentionally low to try and represent the *in vivo* trabecular osteoblast environment.^1^ Other studies investigating the response of IDG-SW3 to mechanical stimuli are thus far limited. They have previously been shown to undergo morphological changes in response to high (1.2 Pa) unidirectional FSS, but not low (0.3 Pa) FSS applied in the same manner, but measures of osteocytogenesis were not reported.^62^ In that morphology study, the low FSS applied where no response was observed was an order of magnitude greater than that exerted here by the see-saw rocker, which may support that the stimuli were too low here. To date, there are no other studies that looked to mechanically stimulate IDG-SW3 directly with FSS, although when compressed within a 3D hydrogel, the resulting fluid flow within the gel was hypothesized to be the cause of upregulated prostaglandin expression (an approximation of the FSS was not made).^63^ Finally, mechanically stretching IDG-SW3 has been shown to downregulate sclerostin, which has the potential to promote mineralization and therefore further osteocytogenesis.^64^

In summary, biochemical treatment with βGP and AA2P, not the application of low magnitude FSS, regulated bone formation and subsequent osteocytogenesis of IDG-SW3. Specifically, the presence of AA2P increased collagen deposition and ALP activity whilst βGP concentration regulated mineralization and mediated osteocytogenesis. This study reveals that the biochemical pathways involved in bone-mineralization may be more influential in the osteoblast–osteocyte transition than low magnitude FSS, and that the presence of mineralizing osteoblasts is essential when studying this process *in vitro.*
